# Effect of Hot Junction Size on the Temperature Measurement of Proton Exchange Membrane Fuel Cells Using NiCr/NiSi Thin-Film Thermocouple Sensors

**DOI:** 10.3390/mi15111375

**Published:** 2024-11-14

**Authors:** Huijin Guo, Zhihui Liu, Tengda Guo, Yi Sun, Kai Shen, Bi Wang, Yongjun Cheng, Yuming Wang, Tiancai Ma, Zixi Wang, Wanyu Ding

**Affiliations:** 1 School of Automotive Studies, Tongji University, Shanghai 201804, China; 1811472@tongji.edu.cn (H.G.); 1811471@tongji.edu.cn (T.M.); 2 State Key Laboratory of Tribology in Advanced Equipment, Tsinghua University, Beijing 100084, Chinagtd21@mails.tsinghua.edu.cn (T.G.); yi_sun.2000@outlook.com (Y.S.); yumingwang@tsinghua.edu.cn (Y.W.); 3 College of Mechanical Engineering, Dalian Jiaotong University, Dalian 116028, China; 4 Engineering Research Center of Continuous Extrusion, Ministry of Education, Dalian Jiaotong University, Dalian 116028, China; skpb_1995@163.com; 5 State Key Laboratory of High-End Compressor and System Technology, Hefei 230031, China; 18435841752@163.com; 6 Laboratory of Low Frequency Electromagnetic Communication Technology with the WMCRT CSSC, Wuhan 430200, China; 15142394597@163.com; 7 College of Materials Science and Engineering, Dalian Jiaotong University, Dalian 116028, China

**Keywords:** Seebeck effect, thin film thermocouple, proton exchange membrane fuel cell, heat transfer, failure analysis

## Abstract

In the process of using thin-film thermocouples for contact measurement of the reaction temperature in proton exchange membrane fuel cells (PEMFC), the impact of thin-film thermocouple volume on the system’s reaction temperature field variation, reaction efficiency, and the lifespan of thermocouples under these conditions is not thoroughly studied. Using magnetron sputtering technology, NiCr/NiSi thin-film thermocouples (NiCr/NiSi TFTCs) with different junction sizes were fabricated on the proton exchange membrane (PEM). These NiCr/NiSi TFTCs exhibit excellent compactness, with thickness and planar dimensions in the micrometer range. When PEMFCs are equipped with built-in NiCr/NiSi TFTCs of different hot junction sizes, the time required for the system to reach a steady state varies with the size of the hot junction, with smaller hot junction sizes reaching a steady state more quickly. In a 500-h continuous operation test, the failure rates of NiCr/NiSi TFTCs also vary based on the hot junction size. Both smaller and larger hot junction sizes have relatively higher failure rates, whereas medium-sized junctions have a lower failure rate. These extensive and repetitive comparative experiments provide significant reference value for the size design of TFTCs operating inside PEMFCs, promoting both industrial production and scientific research.

## 1. Introduction

Proton exchange membrane fuel cells (PEMFCs) are widely regarded as a promising carbon-neutral energy source due to their high energy conversion efficiency and zero harmful gas emissions [1,2,3,4,5,6,7,8]. They have broad application prospects in public transportation, portable emergency power supplies, and combined heat and power solutions. However, like other fuel cell technologies, PEMFCs face a series of challenges related to insufficient durability before they can be deployed on a large scale commercially. These challenges increase costs and pose safety risks, and one of the main reasons for insufficient durability in PEMFCs is thermal management issues.

Effective thermal management is crucial for the optimal performance and longevity of PEMFCs. One major obstacle to achieving effective thermal management is the difficulty in accurately measuring the internal temperature of PEMFCs. Many researchers have attempted to measure the internal temperature fields of PEMFCs using various methods. For example, Chong Fang Ma et al. [9]. Used infrared imaging technology to measure the temperature field on the anode side surface of a membrane electrode assembly (MEA) under a serpentine flow field in a self-designed PEMFC without humidification, with measured temperatures ranging from 25.6 °C to 27.3 °C. Brian Sangeorzan et al. [10]. Demonstrated preliminary progress in creating a non-invasive temperature measurement system based on fluorescence thermometry, which can also optically detect the formation and movement of liquid water in PEMFC gas channels. Experimental data indicated a relationship between temperature variations in PEMFC gas channels and water droplet movement. Akira Nishimura et al. [11]. Studied the impact of the thickness of the gas diffusion layer (GDL) on heat and mass transfer characteristics and power generation performance of PEMFCs at an operating temperature of 90 °C, using a thermal imager to measure the in-plane temperature distribution of the anode and cathode separators. Ping Wen Ming et al. [12]. Manufactured a robust gold thin-film resistance temperature detector (RTD) based on micro-electro-mechanical systems (MEMS) for internal temperature monitoring in PEMFCs.

Thin-film thermocouples (TFTCs) have emerged as a promising solution for real-time temperature sensing within PEMFCs due to their small size, fast response time, and ability to operate under harsh conditions. TFTCs serve as vital temperature sensors that can provide precise temperature measurements without significantly disturbing fuel cell operation. Various materials have been employed as TFTCs in the literature to enhance their performance and durability. Common material combinations include noble metals like platinum and gold, as well as metal pairs such as nickel–chromium (Ni-Cr) and nickel-silicon (Ni-Si) [13,14,15,16,17,18].

When using metal films for temperature measurement, the lifespan of the film and the response speed are two important indicators for evaluating the performance of a film-based temperature measurement system. Factors affecting the lifespan of the film mainly stem from high-temperature failure. Yang L. and others developed and tested an environmental simulator with high-speed rotation and gas thermal shock characteristics to evaluate the lifespan of thermal barrier coatings under high-temperature conditions [19]. As for the temperature measurement method using TFTCs, many teams have tested and improved the lifespan of TFTCs made of different materials [13,14,15,16,17,18]. Some teams have extended the lifespan of TFTCs by improving their protective layers [20]. Regarding response time, David Kokalj and others designed different protective layers to explore their relationship with TFTC response time [21]. Despite these advancements, the effect of different thermojunction sizes on the response time of TFTCs made from the same material has not been thoroughly studied. Understanding this relationship is crucial for optimizing the design of TFTCs to achieve faster response times without compromising durability. Therefore, the goal of this work is to investigate how various thermojunction sizes affect the response time of TFTCs composed of the same material. By systematically studying the influence of thermojunction size on response time, this research aims to provide valuable insights for the design of more efficient temperature sensors in PEMFCs, contributing to better thermal management and enhanced durability of fuel cells.

## 2. Materials and Methods

### 2.1. Working Principle of Thermocouple

The effect connecting thermal and electrical phenomena is known as the thermoelectric effect, also referred to as the Seebeck effect. The working principle of a thermocouple relies on this Seebeck effect. It involves creating a closed circuit with two conductors of different compositions or crystal structures. When one end is heated, an electromotive force (thermoelectric voltage) is generated between the hot and cold ends, causing current to flow in the circuit. By measuring this voltage, the temperature difference between the hot and cold ends can be determined. The calculation methods are provided by Equations (1)–(3).

(1) $$E_{AB}\left(T,T_{0}\right)=\frac{K}{e}t\int_{T_{0}}^{T}\ln\frac{N_{At}}{N_{Bt}}dt$$

(2) $$E_{AB}=\int_{T_{0}}^{T}\left[S_{B}\left(t\right)-S_{A}\left(t\right)\right]dt=\int_{T_{0}}^{T}S_{AB}\left(t\right)dt$$

In the equations:

*E*_A_(*t*)—Absolute thermopotential rate of electrode A;

*E*_B_(*t*)—Absolute thermopotential rate of electrode B;

*S*_AB_(*t*)—Seebeck coefficient for thermocouples.

When the thermal electrode material is determined, *S*_A_(t) and *S*_B_(t) are constant values, so Equation (2) can be reduced to:

(3) $$E_{AB}\left(t\right)=S_{AB}\left(t\right)\left(T-T_{0}\right)$$

According to Equation (3), the thermoelectric voltage *E*_AB_ is linearly related to the hot end temperature *T*. Therefore, by using this linear relationship and assuming the cold end temperature is known and constant, the temperature at the hot end of the thermocouple can be calculated simply by measuring the output voltage of the thermocouple.

### 2.2. Temperature Measurement Structure of PEM

**Figure 1:**
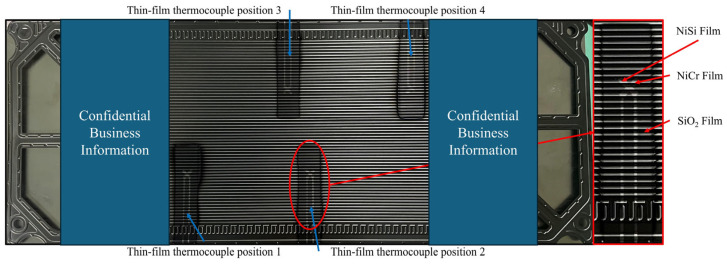
Schematic diagram of the NiCr/NiSi thin-film thermocouple position on the PEM.

NiCr/NiSi TFTCs were fabricated on the surface of the PEM of a commercial PEMFC. The PEM channels are made of stainless steel with a width of 2 mm and a depth of 1 mm. The overall dimensions are 396 × 153 mm^2^. The positions of the NiCr/NiSi TFTCs on the PEM are shown in Figure 1, with two pairs of NiCr/NiSi TFTCs placed in the middle of the PEM and another two pairs placed at the edges of the PEM. Four NiCr/NiSi TFTCs are arranged in a centrosymmetric pattern on the PEM.

### 2.3. Preparation and Calibration of NiCr/NiSi Thin Films

NiCr films, NiSi films, and their SiO_2_ protective layers were fabricated using magnetron sputtering. The NiCr and NiSi films had a thickness of 800 ± 50 nm, while the SiO_2_ layer had a thickness of 1000 ± 50 nm. The deposition was carried out in a vacuum chamber using NiCr and NiSi targets with 99.99% purity and a Si target for the SiO_2_ layer. Prior to deposition, the bipolar plates were cleaned with acetone, ethanol, and ultrapure water, followed by drying in a drying chamber to ensure surface cleanliness. Mechanical masks were cleaned using the same procedure. NiCr films were deposited first, followed by a mechanical mask change for NiSi film deposition and another mask change for the deposition of the SiO_2_ layer. Detailed parameters for the preparation of NiCr, NiSi, and SiO_2_ films are provided in Table 1.

First, a layer of SiO_2_ insulating film is deposited on the PEM to obtain a smooth and well-adhered functional film preparation substrate. The size of this insulating substrate is 30 × 70 mm^2^. Then, a layer of NiCr film is deposited on the SiO_2_ insulating film, followed by a layer of NiSi film deposition on this structure, completing the preparation of the functional films. Finally, a layer of SiO_2_ protective film is deposited on the surface of the functional films to encapsulate the entire temperature measurement system, isolating it from external factors such as corrosion and oxidation. The completed NiCr/NiSi TFTC prepared on the surface of the PEM is also shown in Figure 1.

From the same batch of prepared PEM, select one PEM and cut out two areas containing thin-film thermocouples. One area is used for static calibration and the other for dynamic calibration. In this experiment, NiCr and NiSi films are connected and fixed to copper wires using DB5015 conductive silver paste (DBC, Taian, China). DB5015 is chosen for its excellent heat resistance (up to 1200 °C), water resistance, acid resistance, resistance to organic corrosion, and superior conductivity with low impedance. However, it requires preparation before use as follows: At room temperature, measure components A and B in a mass ratio of 2.5:1 and mix them into a flowable paste for use. After cleaning the copper wires and the surfaces of the NiCr and NiSi films, fix the copper wires at the ends of the NiCr and NiSi films using PI tape, ensuring good adhesion. Cover the adhered areas with the prepared paste and let it sit at room temperature for 12–24 h. Then, slowly heat it in a 202-00B electric thermostat drying oven to 80 °C, maintain this temperature for 2 h, and finally allow it to cool slowly back to room temperature before removing it for use.

**Figure 2:**
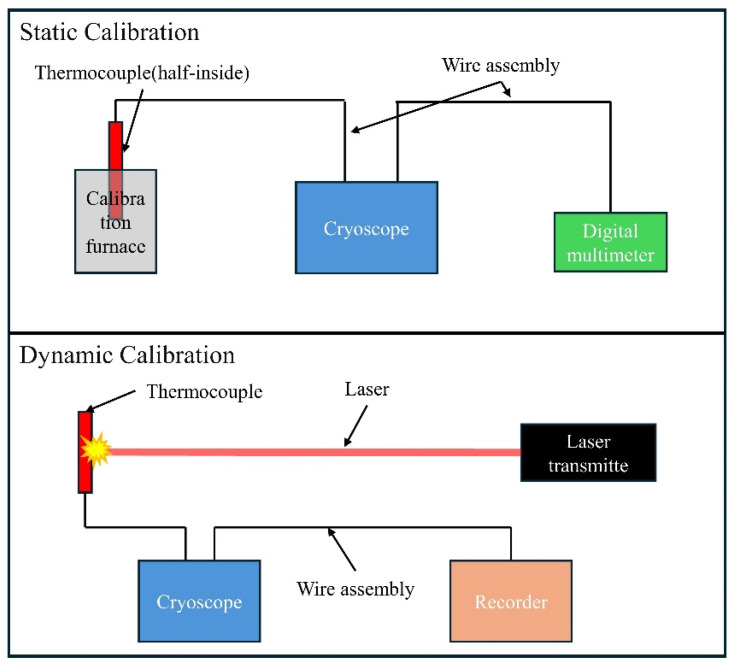
Static and dynamic calibration system structure diagram.

The static calibration system is shown in Figure 2. The bipolar plate samples with thin-film thermocouples are placed in a Fluke 9144 calibration furnace (Fluke, Everett, WA, USA) with asbestos insulation at the furnace mouth. The thermocouple leads are exposed outside the furnace, with one end of the connected copper wire placed in a Fluke 9170 ice point instrument (Fluke, Everett, WA, USA). The ice point instrument is set to zero degrees, and the other end of the copper wire is connected to a DMM 7510 digital multimeter (Keysight Technologies, Santa Rosa, CA, USA). The electromotive force readings on the multimeter are recorded to calculate the Seebeck coefficient. In this study, the set temperature range is 50~505 °C, with eight evenly spaced temperature points, each maintained for five minutes.

Dynamic calibration is performed using a custom dynamic calibration system, as shown in Figure 2. This system uses a Quantel ultra-50 short-pulse infrared laser system, with the laser directed perpendicularly to the thermocouple plane and irradiating the center of the NiCr/NiSi TFTC hot junction. The laser has a wavelength of 1064 nm, a pulse width of 7.6 ns, and a spot diameter of 2.56 mm. Similar to the static calibration system, a Fluke 9170 ice point instrument is used to maintain the cold end temperature around zero degrees. An MR6000 data logger system is connected to the copper wires linked to the NiCr/NiSi thin-film thermocouple to record the thermoelectric voltage signal, with a time resolution of 5 ns and a voltage resolution of 65 μV.

## 3. Results and Discussion

### 3.1. The Temperature Measurement Results of NiCr/NiSi TFTCs with Different Hot Junction Sizes

First, it should be noted that the rate of heat conduction is constant between materials of the same kind, and the heat absorbed by the material (*Q*) is determined by Equation (4) [22].

(4) Q=mcΔT

For the same material, its specific heat capacity (*c*) is identical, meaning that the heat required for a change in temperature depends only on its mass. According to Equation (5), it is evident that for the same material, the density (*ρ*) is also identical, which implies that the required heat depends solely on the geometric dimensions of the material.

(5) m=ρV

**Figure 3:**
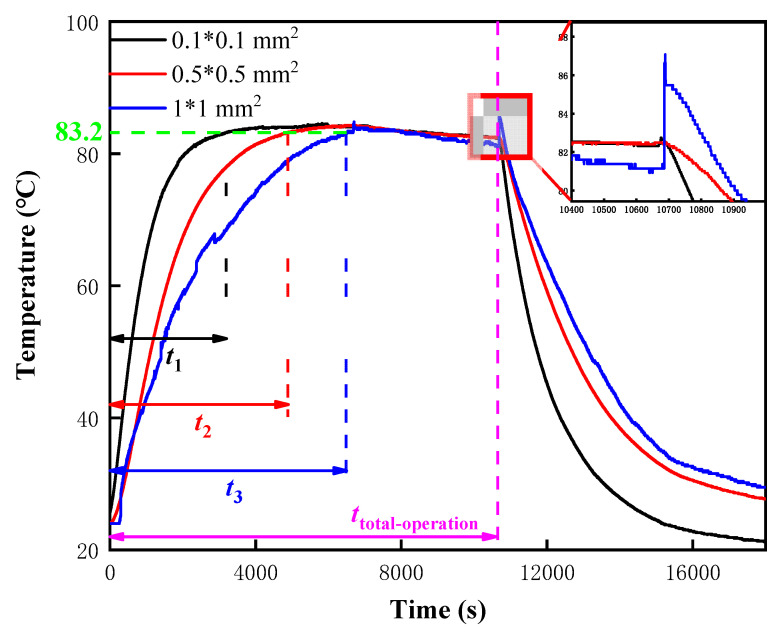
The effect of different hot point two-dimensional sizes on the response time and temperature measurement accuracy of NiCr/NiSi TFTCs temperature measurement systems.

As the hot junction size of the NiCr/NiSi TFTCs increases, the time for the system to transition from startup to a stable operating state also increases, as shown in Figure 3. When the hot junction size is 0.1 × 0.1 mm^2^, the time taken for the system to stabilize is *t***_1_** = 3200 s, with the specific temperature curve shown as the black line in Figure 3. Through repeated experiments, it was found that the stable operating temperature of this self-built system is T = 83.2 °C. Therefore, it can be considered that the system has reached a stable operating temperature once the temperature reaches 83.2 °C. In Figure 3, similar to the representation for the 0.1 × 0.1 mm^2^ size, the stabilization time for the 0.5 × 0.5 mm^2^ size is *t*_2_ = 4900 s, shown as the red line in the figure. For the 1 × 1 mm^2^ size, the stabilization time is *t*_3_ = 6500 s, indicated by the blue line in Figure 3.

After the system has been operating for *t*_total-operation_ = 10,700 s and is then shut down, it can be observed that the temperature of the NiCr/NiSi TFTCs drops rapidly after the reaction stops. However, due to the different thermal melting effects of the various hot junction volumes, the black line, which represents the smallest area, drops the fastest. The red line, representing the medium area, and the blue line, representing the largest area, have relatively similar descent rates, with the red line being slightly faster. Similarly, during the heating process, the different stabilization times of the three NiCr/NiSi TFTCs with different hot junction sizes are primarily due to the differences in their heating rates, which are caused by their varying thermal melting properties.

As for the temperature fluctuations within the red rectangle in Figure 3, these are due to the sudden shutdown of the PEMFC, causing dramatic changes in the PEM surface environment and resulting in a rapid temperature drop. For NiCr/NiSi TFTCs, this places them in an unstable temperature field with a significant temperature difference between the upper and lower surfaces. In recent studies, Sun et al. [23] have consistently observed significant signal fluctuations of NiCr/NiSi TFTCs in unstable temperature fields and have thus developed a dynamic heat transfer mathematical model for NiCr/NiSi TFTCs. The temperature fluctuations observed here can be fully explained by the internal heat transfer and reflection within NiCr/NiSi TFTCs under unstable temperature fields, leading to localized temperature increases. This is a very normal and interesting phenomenon.

### 3.2. The Effect of Hot Junction Size on the Lifespan of NiCr/NiSi TFTCs

**Figure 4:**
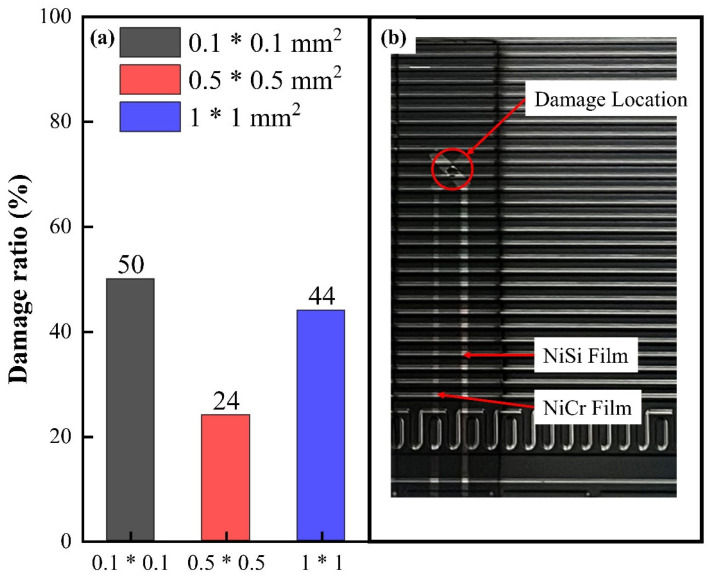
(**a**) The ratio of failed NiCr/NiSi TFTCs to the total number for each hot junction size. (**b**) The damage location diagram of failed NiCr/NiSi TFTCs.

The experiment on the effect of hot junction size on the lifespan of NiCr/NiSi TFTCs is shown in Figure 4. In this experiment, 50 PMCs were used to test the lifespan of NiCr/NiSi TFTCs. Each thermocouple operated in the PEMFC for 500 h, and the relevant data were recorded. The damage condition of two pairs of NiCr/NiSi TFTCs located in the middle of the PMC, as shown in statistical Figure 1, was assessed. These two pairs are in the same position within the PMC, providing a strong basis for comparison. The failure of NiCr/NiSi TFTCs is evaluated based on the following three tests, where failure in any one of these tests indicates that the NiCr/NiSi TFTCs are damaged and cannot continue to operate normally: (1) The structure of NiCr/NiSi TFTCs shows visible cracks and peeling of the thin film, as shown in Figure 4b. (2) The electrical resistance between the functional film (NiCr or NiSi) leads and the PMC surface is less than 1 × 10^5^ Ω. (3) Damage at the connection between NiCr/NiSi TFTCs and the leads.

From Figure 4a, it can be seen that when the hot junction size is 0.1 × 0.1 mm^2^, 50% of 100 pairs of NiCr/NiSi TFTCs are judged to be damaged. When the hot junction size is 0.5 × 0.5 mm^2^, 24% of 100 pairs of NiCr/NiSi TFTCs are judged to be damaged. Similarly, when the hot junction size is 1 × 1 mm^2^, 44% of 100 pairs of NiCr/NiSi TFTCs are judged to be damaged. It can be seen that the damage rate of NiCr/NiSi TFTCs with sizes in the range of 0.1 × 0.1 mm^2^ to 1 × 1 mm^2^ is generally below 50%. There are two main influencing factors. First, as the size decreases, the mechanical strength of the NiCr/NiSi TFTCs reduces, and crystal defects within the thin film are magnified, leading to overall failure. Second, as the size increases, the effect of differing thermal expansion coefficients between the layers of NiCr/NiSi TFTCs becomes more pronounced. In NiCr/NiSi TFTCs, the thermal expansion coefficients of the NiCr and NiSi films are almost identical, but the thermal expansion coefficient of SiO_2_ is significantly different from that of the NiCr/NiSi films. This difference causes slippage between the NiCr/NiSi layer and the SiO_2_ layer under mechanical stress. On a macroscopic level, this can be observed as film fracture due to compressive deformation, as shown in Figure 4b.

### 3.3. Performance Characterization of NiCr/NiSi TFTCs

To explain and investigate the phenomena mentioned in Section 3.1 and Section 3.2, it is necessary to characterize the NiCr film, NiSi film, and SiO_2_ film. This will involve examining the microstructure and performance of NiCr/NiSi TFTCs to elucidate these two phenomena.

**Figure 5:**
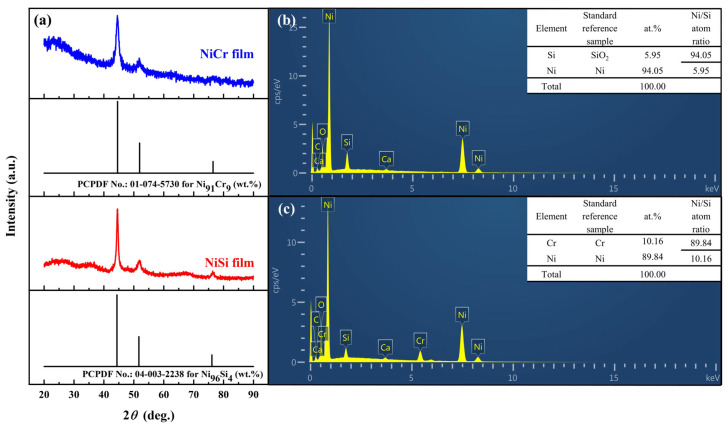
Basic characteristics of NiCr and NiSi thin films: (**a**) XRD patterns of NiCr thin films, NiCr target material, and the standard XRD pattern of Ni_91_Cr_9_ (wt.%); XRD patterns of NiSi thermoelectric electrodes, NiSi target material, and the standard XRD pattern of Ni_94_Si_6_ (wt.%). (**b**) EDS spectrum of the NiCr thin film. (**c**) EDS spectrum of the NiSi thin film.

Figure 5a displays the XRD patterns of the NiCr and NiSi thin films. To verify the compactness of the NiCr and NiSi thin films, EDS measurements were performed, as shown in Figure 5b,c. Only C and O elements were detected as impurities, which is attributed to contamination from organic substances in the environment during sample transfer since EDS measurements are not conducted in situ. The EDS results reveal that the NiCr thin film is composed of Ni and Cr elements with an atomic ratio of Ni/Cr = 89.84/10.16, and the NiSi thin film is composed of Ni and Si elements with an atomic ratio of Ni/Si = 94.05/5.95. These results are consistent with the composition of the target material and the standard K-type thermocouple thermoelectric electrodes [24,25]. This indicates that the films are dense, and there are no macroscopic defects caused by the preparation process. However, it does not prevent macroscopic film damage caused by lattice defects, which remains a challenging issue to address [26,27].

The static and dynamic calibration results of NiCr/NiSi TFTCs with different hot junction sizes are shown in Table 2. It can be seen from Table 2 that the change in hot junction size does not affect the Seebeck coefficient of the NiCr/NiSi TFTCs, which is completely consistent with the definition of the Seebeck coefficient. However, the dynamic calibration results show a strong correlation with the hot junction size, which is also understandable. In dynamic calibration, the hot junction is in an unstable temperature field, and the dynamic calibration results are greatly influenced by the thermal melting of the metal at the hot junction. In other words, under the same conditions, the response time depends on the size of the thermal melting at the hot junction of the NiCr/NiSi TFTCs.

### 3.4. The Effect of NiCr/NiSi TFTCs Hot Junction Size on the Operating State of PEMFCs

**Figure 6:**
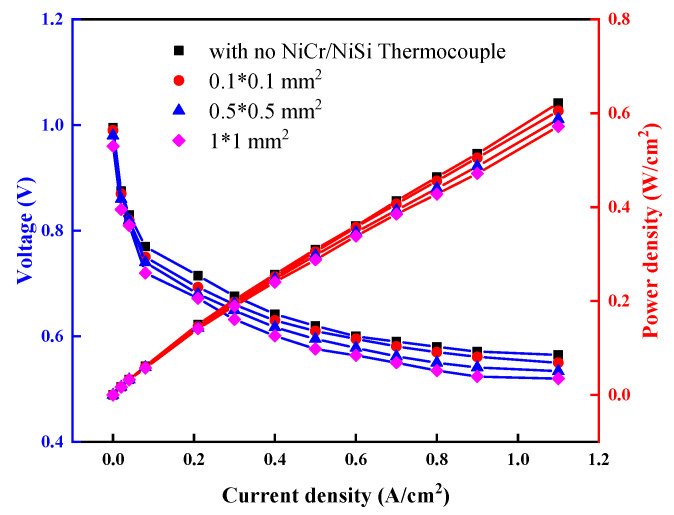
The effect of NiCr/NiSi TFTCs with different hot spot sizes on the I-V and I-P polarization curves of PEMFC.

Figure 6 presents the I-V and I-P polarization curves of PEMFCs under specific conditions, comparing three different hot junction sizes of NiCr/NiSi TFTCs and a PEMFC without the NiCr/NiSi functional film, all using the same size of the SiO_2_ insulation layer. The conditions include an H_2_/air input pressure of 0.4 MPa, a flow rate of 500 mL/min, an inlet relative humidity of 40%RH, an ambient temperature of 25 °C, and a stable operating temperature of the PEMFC at 83.2 °C. Figure 6 demonstrates that coating the PEM surface with NiCr/NiSi TFTC measurement devices has a minimal impact on the I-V polarization curves. As the hot junction area of the NiCr/NiSi TFTCs increases, there is a slight voltage drop at the same current density. When the current density exceeds 0.1 A/cm^2^, the output voltage of PEMFCs with NiCr/NiSi TFTCs decreases with increasing hot junction volume, but the differences remain within 5%, which is acceptable. Similarly, comparing the effects of different NiCr/NiSi TFTCs hot junction sizes on the I-P curves further demonstrates that changes in the hot junction size of NiCr/NiSi TFTCs have no significant impact on power output. The difference in power output between the group without NiCr/NiSi thin-film thermocouples and the group with NiCr/NiSi TFTCs having a hot junction area of 1 × 1 mm^2^ at a current density of 1.1 A/cm^2^ is only Δ*P* = 0.05 W/cm^2^. It can be observed that, although the NiCr/NiSi TFTCs coating on the PEM surface slightly hinders proton exchange and heat transfer processes in the coated area, this effect is negligible.

### 3.5. Optimal Design of NiCr/NiSi TFTCs Hot Junction Size

To better explore the optimal balance between the lifespan and performance of NiCr/NiSi TFTCs with different junction sizes, a benefit evaluation criterion was designed and constructed to provide a quantitative assessment standard. The calculation method is as follows:

(6) $$R_{i}=P_{i}\left(1-F_{i}\right)$$

where *R_i_* represents the benefit of the *i*-th junction area of NiCr/NiSi TFTCs, *P_i_* represents the performance index of the *i*-th junction area of NiCr/NiSi TFTCs, and *F_i_* represents the failure rate of the *i*-th junction area of NiCr/NiSi TFTCs. Where the performance index *P_i_* is obtained by the following formula:

(7) $$P_{i}=\frac{1000}{t_{i}}\cdot 100$$

Equation (6) can be arranged as follows:

(8) $$R_{i}=\frac{1000}{t_{i}}\cdot 100\cdot \left(1-F_{i}\right)$$

**Figure 7:**
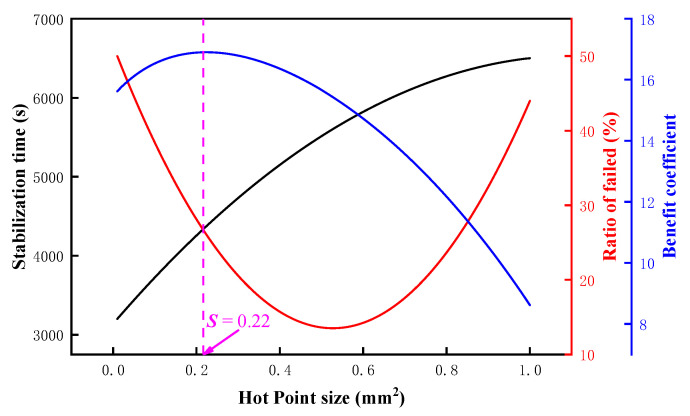
The calculation results of the benefit coefficient for different junction sizes.

Using a quadratic polynomial, fit the failure rate data from Figure 4a and the stabilization time data from Figure 3. Supplement the complete data for the junction area of NiCr/NiSi TFTCs within the range of 0.1 × 0.1 mm^2^ to 1 × 1 mm^2^ with a step size of 0.01 mm^2^ for both *t* and *F*. A total of 91 junction sizes were calculated, and the results are shown in Figure 7. When the junction area *S* = 0.22 mm^2^, the benefit coefficient reaches its maximum value. This indicates that the optimal junction size for NiCr/NiSi TFTCs is 0.47 × 0.47 mm^2^.

## 4. Conclusions

Designed experiments and discussed the impact of the hot junction size of NiCr/NiSi TFTCs on the performance of the temperature measurement system in PEMFCs and the working efficiency of PEMFCs. By designing three different hot junction sizes—0.1 × 0.1 mm^2^, 0.5 × 0.5 mm^2^, and 1 × 1 mm^2^—the comprehensive performance of different hot junction sizes is evaluated using two parameters: the time required for system stabilization and the damage rate of NiCr/NiSi TFTCs after 500 h of operation. The experiment revealed that as the hot junction size increases, the stabilization time also increases. Furthermore, as the hot junction size increases from 0.1 × 0.1 mm^2^ to 1 × 1 mm^2^, the damage rate of NiCr/NiSi TFTCs initially decreases and then increases. This is consistent with the amplification rule of lattice defect effects at the microscale and the positive correlation between thermal expansion and object volume. The benefit evaluation algorithm determined that the optimal hot junction size for NiCr/NiSi TFTCs is 0.47 × 0.47 mm^2^. This has significant reference value and serves as a driving force for establishing standards for the size of thin-film thermocouples (TFTCs) used in measuring PEMFCs. It also provides a basis for studying the damage mechanisms of TFTCs under extreme conditions. In the future, we will continue to optimize the algorithm and conduct supplementary tests to determine the optimal size of NiCr/NiSi TFTCs. Additionally, we will extend the conclusions to TFTCs made from different material compositions.

## Figures and Tables

**Table 1:** The deposition parameters for NiCr, NiSi, and SiO_2_ films.

Experimental Parameters	NiCr Film	NiSi Film	Sio_2_ Film
Target (wt.%)	Ni_90_Cr_10_	Ni_97_Si_3_	Si
Target purity	99.9%	99.9%	99.99%
Target base distance (mm)	120	120	120
Working gas	Ar	Ar	Ar/O_2_
Working pressure (Pa)	0.7	0.7	0.6
Flow rate (sccm)	20	20	20/10
Inversion time (μs)	1	1	1
Pulse frequency (kHz)	100	100	100
Sputtering power density (W/cm^2^)	1.90	1.90	3.33
Film thickness (nm)	800 ± 50	800 ± 50	1000 ± 50
Sputtering time (min)	40	45	90

**Table 2:** Static and dynamic calibration results for different thermocouple junction sizes.

Hot Point Size	Static Calibration (μV/°C)	Dynamic Calibration (μs)
0.1 × 0.1 mm^2^	10.4	0.53
0.5 × 0.5 mm^2^	10.2	1.05
1 × 1 mm^2^	10.3	2.33

## Data Availability

The original contributions presented in the study are included in the article, further inquiries can be directed to the corresponding authors.
